# Hit me with your best puff: Personality predicts preference for cigar vs. cigarette smoking

**DOI:** 10.1371/journal.pone.0305634

**Published:** 2024-07-03

**Authors:** Dritjon Gruda, Jim A. McCleskey

**Affiliations:** 1 Catolica Porto Business School, Research Centre in Economics and Management, Universidade Catolica Portuguesa, Lisboa, Portugal; 2 School of Business, Maynooth University, Maynooth, Ireland; 3 Western Governors University, Millcreek, Utah, United States of America; Imperial College London, UNITED KINGDOM

## Abstract

In this study, we examine the association between Big Five personality traits and cigar or cigarette smoking in a sample of 9,918 older adults across 11 European countries derived from the Survey of Health, Ageing and Retirement in Europe (SHARE) dataset. We find significant associations between several traits and smoking groups. Smoking was associated with lower scores on Conscientiousness and Agreeableness and higher Extraversion scores. In addition, cigar smokers exhibit lower Neuroticism and higher Openness compared to both cigarette smokers and non-smokers. These findings suggest that both personality traits are antecedents of smoking behavior, offering implications for targeted public health interventions and social policies aimed at combating the global tobacco epidemic.

## Introduction

Tobacco use remains a formidable global public health challenge, responsible for over 8 million deaths annually, including those attributed to second-hand smoke exposure [1]. The burden is particularly heavy in low- and middle-income countries, where the economic and health costs of tobacco consumption are profound. While the physiological risks of tobacco use, such as cardiovascular diseases and cancer, are well-documented [2], emerging research underscores the critical role of psychological factors, including personality traits, in shaping tobacco consumption patterns [3, 4].

The Big Five personality framework, encompassing Openness, Conscientiousness, Extraversion, Agreeableness, and Neuroticism, offers a robust lens through which to examine individual behavioral tendencies across cultures [5], including those related to substance use. Research has consistently demonstrated that certain personality traits, such as high Neuroticism and low Conscientiousness, are predictive of smoking behaviors, suggesting a predisposition towards tobacco use among individuals with these personality profiles [3, 6, 7]. Specifically, elevated Neuroticism has been linked to smoking initiation and persistence, potentially due to its association with stress and anxiety relief. Conversely, lower levels of Conscientiousness may reflect a propensity for impulsivity and risk-taking, factors known to contribute to the initiation of smoking. While Extraversion’s role is more nuanced, it is generally associated with social smoking and the initiation phase, possibly due to the social nature of smoking in certain contexts [8, 9].

In this paper, we posit that it is unlikely that the relationship between personality traits and smoking is uniform across all tobacco products. Different forms of tobacco usually are associated with varying rituals, social implications, and potential motivations. For instance, while cigarette smoking might be a habitual response to daily stressors, cigar smoking could be an occasional indulgence tied to social prestige or cultural practices [10]. In support of this notion, preliminary studies suggest that the personality profiles of individuals who prefer cigarettes may differ from those who choose other tobacco forms, such as cigars or smokeless tobacco [11]. This variance underscores the need for a more granular examination of how personality influences tobacco consumption choices. Understanding these distinctions is crucial for developing targeted interventions that address the specific motivations and vulnerabilities of different smoker groups [12], thereby enhancing the effectiveness of public health campaigns aimed at reducing tobacco use. Moreover, the cultural context of tobacco use cannot be ignored, as cigar smoking is more prevalent and even on the rise in some cultures. For example, data from the Observatory of Economic Complexity [13] indicated that the top 10 cigar-importing nations in the world in 2022 were the US, Germany, Italy, France, Spain, Switzerland, Belgium, the UK, the Netherlands, and Greece in that order. Hence, Europe is well-represented among cigar-consuming nations.

In addition, conducting this research across multiple countries is vital for capturing the different ways in which cultural contexts influence the interplay between personality traits and tobacco use. Tobacco consumption preferences and associated personality traits that underpin them can vary significantly from one cultural setting to another [14–16]. Addressing the global tobacco crisis requires analyzing data beyond a single nation to understand how personality influences tobacco use. This study, with 9,918 participants from 11 countries, aims to provide these much-needed additional insights.

### A smoking personality

The idea that various personal characteristics can forecast one’s health, health-related behaviors, and overall wellness is a fundamental concept across several fields such as medicine, psychiatry, health psychology, and clinical psychology [17]. This concept also acts as a significant driving force behind personality theory and research. For example, Atherton, Robins [18] found that personality is associated with risky health outcomes, namely self-reported health, body mass index, and substance use in a large sample (N = 460,172). Additionally, Gruda, Hanges [19] found that population-level personality traits, in particular narcissism, are associated with state-level health outcomes. Additionally, Abdullahi et al. [20] suggested that subjective well-being (SWB) and its related positive health outcomes could be enhanced using interventions designed based on individual personality traits. Therefore, it might be warranted to examine the link between personality as a predictor not only as a predictor of smoking behavior (i.e., whether an individual chooses to smoke or not) but more specifically smoking preferences (i.e., how an individual chooses to consume tobacco).

Smoking behavior and its associated factors have long been a subject of scientific inquiry. Among various factors that contribute to smoking habits, the Big 5 personality traits have gained considerable attention. Neuroticism in particular has emerged as one of the most robust predictors of smoking behavior in general [4, 8, 9, 21, 22], with numerous studies indicating that neurotic individuals are more likely to take up smoking and have greater difficulty quitting the habit [e.g., 23]. Highly neurotic individuals often struggle with emotional regulation and may resort to smoking for anxiety and stress relief [22, 24]. Prior studies also indicated a relationship between extraversion and smoking, suggesting that the strongest correlations between personality traits and present smoking habits were predominantly found in neuroticism and extraversion [8, 21].

Conversely, high levels of Conscientiousness—indicative of self-discipline and a focus on long-term goals—are associated with lower rates of smoking initiation and increased likelihood of successful cessation [25]. Agreeableness, a trait representing compassion and cooperativeness, also tends to correlate negatively with smoking. Individuals with low levels of Agreeableness are often more likely to start smoking and less likely to quit, possibly due to reduced interpersonal sensitivity and a lower tendency to comply with social norms that discourage smoking [26]. This is a noteworthy oversight, given that cigar smokers may exhibit distinct personality profiles compared to cigarette smokers [10].

Collectively, while existing research on the Big Five personality traits and smoking behavior has yielded significant insights, it has often overlooked the nuances of individual tobacco product preferences, especially between cigars and cigarettes. To deepen our understanding of how personality traits influence smoking habits, it is crucial to move beyond the simplistic binary of smokers versus non-smokers and explore the subtle differences within these categories. This study specifically investigates the personality profiles of the two predominant smoker groups: cigarette and cigar smokers.

## Methodology

Data for the present study was obtained from the Survey of Health, Ageing, and Retirement in Europe (SHARE) is a multidisciplinary, multi-wave, and cross-national research project conducted across Europe (incl. Israel) with participants aged 50 years and above. The SHARE data set [27] is well suited for this study, as focusing on an older population provides valuable insight into long-term smoking habits and the interplay of personality traits, culture, and smoking behavior.

The dataset is publicly available, has received ethics approval, and informed consent was obtained from all participants. We primarily relied on data collected between 2004 and 2017. Data was collected using computer-assisted personal interviewing (CAPI). SHARE interviewers conduct face-to-face interviews using a laptop on which the CAPI instrument is installed. All analyses were conducted using Stata 17.0.

Based on individuals who indicated both specific tobacco smoking preferences (e.g., cigars, cigarettes, and pipe), and Big Five personality trait scores, we derived a final sample comprised of 9,918 participants (42.53% female) from 11 countries (M_age_ = 61.24, SD = 8.04). Of this sample, 5,480 participants (55.25%) reported they had never smoked (i.e., non-smokers), while 4,438 participants indicated they smoke or had smoked regularly (at some point in their lives, i.e., smokers). The average number of years of smoking across all tobacco users was 26.06 (SD = 13.30). Importantly, while some participants reported they smoked several different types of tobacco, the reported results pertain only to consumers of an exclusive tobacco group (e.g., only cigarettes, or only cigars). Data on the use of electronic cigarettes/heat-not-burn/ nicotine pouches/waterpipe was not available in this sample and is beyond the scope of this study as the question at issue here is specific to cigarettes and cigars.

### Measures

#### Tobacco preference

Participants were asked whether they have ever smoked (0 = never, 1 = smoked), and which tobacco product they have smoked in the past (incl., cigars and cigarettes). Tobacco preference was assessed using a binary variable (0 = not selected, 1 = selected) for cigars and cigarettes. Whenever participants indicated that they had smoked any of the provided tobacco products, they were categorized as “Smokers”. No products selected indication was coded as “Non-Smokers”.

#### Big Five personality traits

Big Five personality traits were measured using the BFI-10 (Rammstedt & John, 2007). Trait-level scores are provided in the SHARE data sets, and include Openness to Experience (M = 3.19, SD = 0.95), Conscientiousness (M = 4.12, SD = 0.78), Extraversion (M = 3.45, SD = 0.92), Agreeableness (M = 3.76, SD = 0.81) and Neuroticism (M = 2.68, SD = 1.04). Example items include I see myself as someone who… “has an active imagination” (Openness to Experience), “does a thorough job” (Conscientiousness), “is outgoing, sociable” (Extraversion), “is generally trusting” (Agreeableness), and “gets nervous easily” (Neuroticism).

#### Demographics and controls

We accounted for age and sex (male, female).

## Results

The examination of correlations between the Big Five personality traits and distinct smoking habits within a sizable sample of 9,818 participants revealed significant findings, as summarized in Table 1. A country-level breakdown of our data is provided in Table 2.

**Table 1:** Pairwise Pearson correlations between variables.

		1	2	3	4	5	6	7	8	9	10
1	Openness										
2	Conscientiousness	0.12***									
3	Extraversion	0.14***	0.18***								
4	Agreeableness	0.01	0.16***	0.18***							
5	Neuroticism	-0.07***	-0.12***	-0.24***	-0.24***						
6	Cigarettes	-0.01	-0.06***	0.02*	-0.03**	-0.00					
7	Cigars	0.01	-0.00	0.01	-0.00	-0.05***	-0.10***				
8	Smoker	0.01	-0.06***	0.04***	-0.03**	-0.05***	0.89***	0.14***			
9	Non-Smoker	-0.01	0.06***	-0.04***	0.03**	0.05***	-0.89***	-0.14***	-1.00		
10	Age	-0.09***	-0.05***	-0.04***	0.04***	-0.04***	-0.12***	0.00	-0.10***	0.10***	
11	Sex	0.04***	0.03*	0.03**	0.05***	0.14***	-0.17***	-0.12***	-0.28***	0.28***	-0.05***

Note: Sex coded 1 (male) and 2 (female)

* *p* < .05

** *p* < .01

*** *p* < .001; n = 9,918.

**Table 2:** Sample characteristics by country.

Country	Participants	Female Population (%)	Age (M)	Age (SD)	Smoking (%)
Austria	491	61.91	62.65	7.90	33.40
Belgium	1434	56.07	61.41	8.34	46.30
Denmark	661	54.77	60.41	8.40	58.09
France	890	59.66	61.15	8.34	40.90
Germany	584	54.79	61.16	7.11	41.95
Greece	1505	57.41	59.69	8.19	45.71
Israel	951	59.20	59.65	7.57	41.53
Italy	1080	56.48	61.71	7.12	45
Spain	859	60.77	63.03	8.29	35.62
Sweden	1085	56.13	62.22	7.69	54.19
Switzerland	378	55.82	62.35	8.41	40.74

Note: n = 9,918.

Consistent with prior research [7–9], a robust correlation was observed between current smoking and lower trait Conscientiousness, particularly evident in cigarette smokers as opposed to cigar smokers. Conversely, smoking exhibited a significant association with heightened Extraversion and diminished Agreeableness, albeit these associations were primarily observable in cigarette smokers. Notably, cigar smoking was negatively associated with Neuroticism.

**Fig 1:**
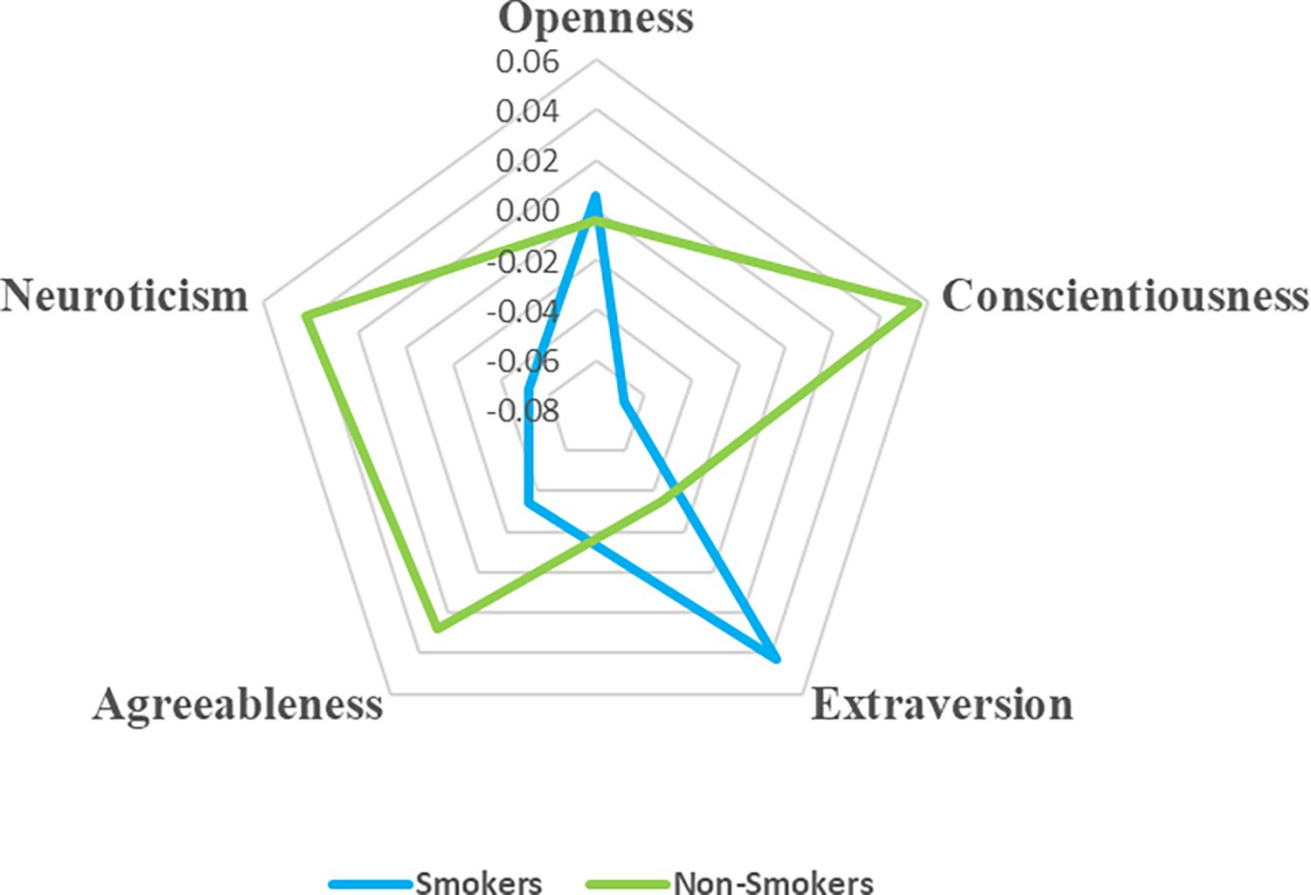
Personality profiles for smokers vs. non-smokers.

**Fig 2:**
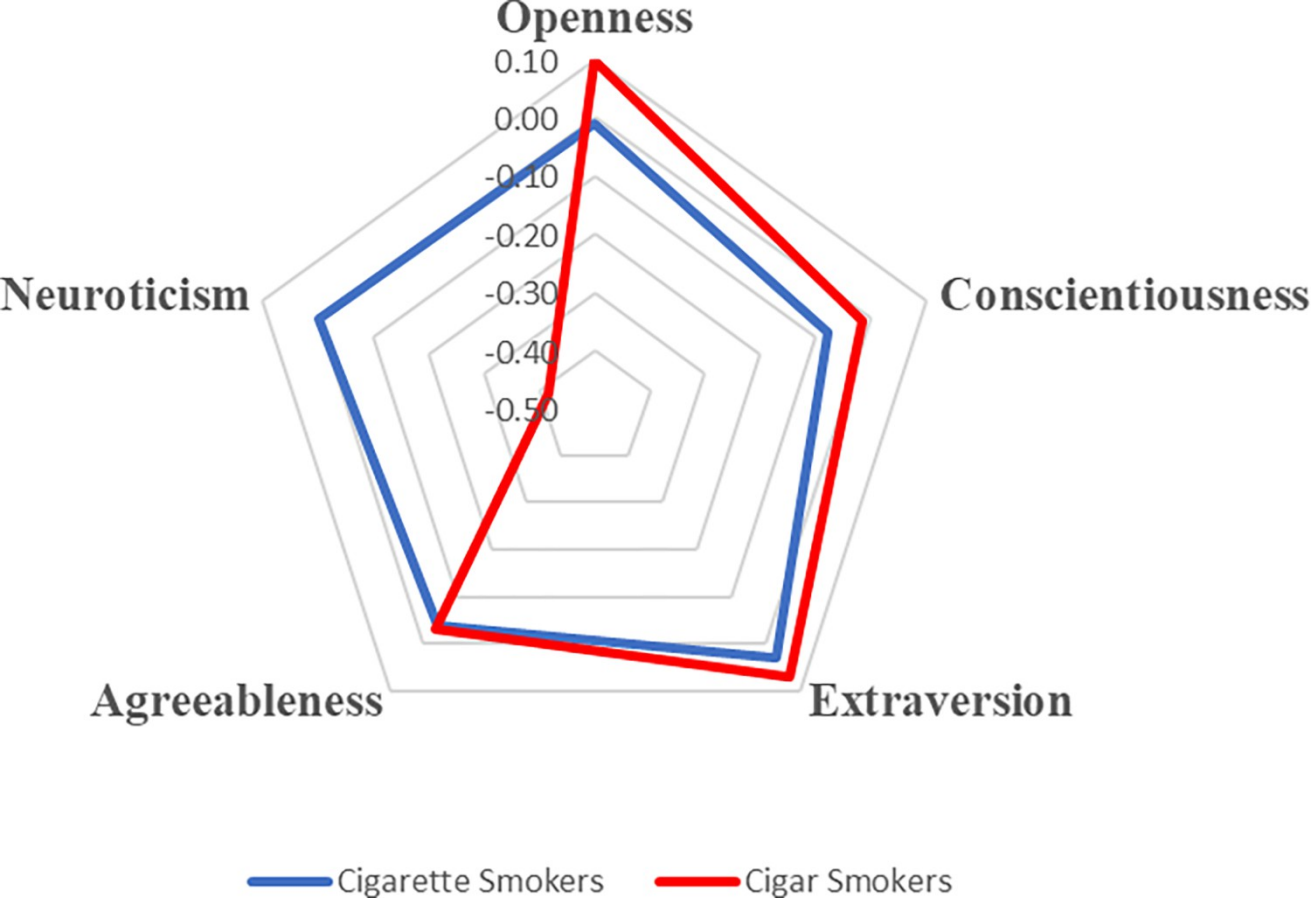
Personality profiles for cigar vs. cigarette smokers.

To visualize these correlations and delineate personality profiles based on tobacco preference, all Big Five traits were standardized (i.e., rescaled to have a mean of zero and a standard deviation of one), resulting in the generation of radar charts for comparative analysis, as depicted in Figs 1 and 2.

Cigar smokers showcased elevated scores in Openness to Experience (M = 0.10), contrasting with the near-zero mean score among non-smokers (M = -0.004). However, neither Openness nor Agreeableness exhibited statistically significant relationships across the smoking categories, except for a modest correlation between Agreeableness and smoking in general (r = −0.03, *p* < .01).

Conscientiousness exhibited a negative correlation across all smoking categories: cigarette smoking (r = −0.061, *p* < .001), cigar smoking (r = −0.002, *p* = .830), and smoking (r = −0.06, *p* < .001), suggesting that individuals with higher conscientiousness levels are less inclined towards smoking behaviors, though the effect sizes were modest and the association with cigar smoking lacked statistical significance. Intriguingly, Extraversion demonstrated a positive correlation with smoking in general (r = 0.04, *p* < .001), albeit insignificantly with specific types of smoking, implying that smokers, regardless of tobacco type, tend to exhibit greater extroversion than non-smokers. Neuroticism displayed a negative correlation with cigar smoking (r = −0.05, *p* < .001), while its correlation with cigarette smoking was negligible (r = −0.001, *p* = .90).

Further investigation entailed contrasting the two predominant smoker groups (i.e., cigar and cigarette smokers) across countries via a multi-level logistic regression with heteroscedastic robust standard errors, the outcomes of which are delineated in Table 3.

**Table 3:** Multilevel regression results predicting exclusive cigar (vs. cigarette) smoking preference.

	b	SE	z	95% CI
Openness to	0.19*	.09	2.14	[.016, 0.36]
Conscientiousness	-0.14	.18	-0.76	[-.50, 0.22]
Extraversion	-0.17	0.09	-1.84	[-.35, 0.01]
Agreeableness	0.03	0.13	0.25	[-.22, 0.28]
Neuroticism	-0.41***	0.12	-3.49	[-.64, 0.18]
Age	-0.00	0.02	-0.20	[-.04, 0.03]
Gender	-1.97***	0.37	-5.28	[-2.70, -1.24]
General Health	-0.04	0.18	-0.23	[-0.39, 0.31]
Years Smoking	0.02	0.02	1.02	[-.02, 0.06]
Constant	-0.11	1.90	-0.06	[-3.83, 3.60]

Note: unstandardized coefficients with heteroscedastic robust standard errors; gender: 1 (male), 2 (female), CI = Confidence Interval

* *p* < .05

** *p* < .01

*** *p* < .001; n = 2181.

The results unveiled that in comparison to cigarette smokers, cigar smokers demonstrated a heightened propensity to exhibit Openness to Experience (b = 0.19, SE = 0.09, *z* = 2.14, *p* = 0.032) and a diminished likelihood of neuroticism (b = -0.41, SE = 0.12, *z* = -3.49, *p* < 0.001). In other words, cigar smokers exhibited a 21% greater likelihood (OR = 1.21) of scoring high on Openness to Experience and a 34% decreased likelihood (OR = 0.66) of being neurotic. Additionally, cigar smokers displayed an 86% higher likelihood of being male than female (OR = 0.14).

## Discussion

The findings of this study illuminate the intricate relationship between the Big Five personality traits and tobacco use preferences among older adults across a diverse set of countries. Overall, we found that individuals who smoke tend to exhibit lower levels of Conscientiousness and Agreeableness and higher levels of Extraversion. The lower Conscientiousness among smokers may reflect a lack of self-discipline and disregard for long-term health risks, characteristic of more impulsive behaviors. Reduced Agreeableness could indicate a tendency towards nonconformity and antagonism, as smoking often persists despite societal disapproval. Conversely, the higher Extraversion observed may be due to the social nature of smoking, which serves as a conduit for social interactions and stimulation, traits favored by extraverted individuals. These correlations suggest that smoking behaviors are closely linked with specific personality dimensions, highlighting how lifestyle choices can both influence and reflect underlying personal characteristics.

A key contribution of this paper, however, was the differentiation between different forms of tobacco consumption, namely cigarette and cigar smokers, and associations between these groups and specific traits. As far as we know, this is the first paper to associate personality traits with smoking behavior while differentiating between smoking preferences. Our analysis revealed that cigar smokers tend to exhibit higher levels of Openness to Experience and lower levels of Neuroticism compared to cigarette smokers, a distinction that underscores the interplay between personality and smoking behavior. The association between higher Openness to Experience and cigar smoking may reflect the cultural and social dimensions of cigar use, often perceived as a symbol of sophistication and leisure. This contrasts with cigarette smoking, which is more commonly linked to stress relief and habit [4]. Such distinctions suggest that the motivations and contexts of tobacco use are as varied as the individuals partaking in these behaviors, pointing to the need for smoking cessation interventions that are not only tailored to the individual’s personality but also consider the social and cultural connotations of different tobacco products.

While the consumption of cigars traditionally has been associated with the behavior of older men, the cigar market is alive and well with a worldwide revenue of US$23.4 billion in 2024 and is on track to grow 4.3% annually from 2024 to 2027 [28]. Premium cigar smoking is typically associated with more affluent and higher social status groups, special occasions, or celebratory events [10]. In such contexts, cigar smoking can be seen as a symbol of prestige and luxury, rather than a means to cope with negative emotions or stress. Additionally, the act of smoking a cigar is often more ritualistic and leisurely, involving a slower pace, less inhaling, and more time to savor the experience. This contrasts with the often more habitual, quick-paced, and compulsive nature of cigarette smoking; behaviors which in turn are associated with personality traits such as higher Neuroticism and lower Conscientiousness.

From a public health perspective, these findings underscore the potential for leveraging personality insights to design more personalized and effective smoking cessation programs. There have been consistent appeals for the inclusion of personality information in health behavior interventions [29–31], precisely because personality is associated with smoking behavior [8]. Specifically, providing increased attention and support to individuals high on the personality dimension of neuroticism could enhance the outcome of smoking cessation interventions. Prior research indicates that interventions targeted at adolescents who exhibit high anxiety sensitivity and hopelessness (i.e., high neuroticism) may effectively prevent and reduce problematic drinking [32]. The findings imply that this could also be applicable to interventions promoting smoking cessation. Additionally, as neuroticism is correlated with depressive symptoms [6] and depression commonly coexists with smoking [33], personality-informed interventions to reduce smoking could also benefit individuals with depressive symptoms.

While a previous study examined the relationship between the Big Five personality traits of practitioners and the rates of smoking cessation of their patients [34], a relative paucity of studies addressed the use of personality-based interventions aimed at reducing tobacco use. For example, Jin et al. [35] found that among Chinese men, reduced Neuroticism, decreased Extraversion, and increased Openness to Experience were linked with higher rates of quitting smoking. Additionally, lower Conscientiousness was associated with successful smoking cessation among Chinese women. These findings underscore the importance of incorporating personality traits into smoking cessation programs, to which our study contributes as well. In another study, Debenham et al. [36] examined a tobacco use prevention program in a large sample of teenagers (13–14 years old) in Australia. The personality-focused program significantly decreased the likelihood of tobacco use and intentions to use tobacco three years after its implementation. These lasting benefits endorse the personalized, trait-based approach to smoke cessation programs. For example, interventions targeting cigar smokers might focus on the social aspects of smoking, offering alternative means of social engagement, or addressing the desire for status or luxury that cigars might represent. Conversely, programs for cigarette smokers might prioritize stress management techniques, aiming to address the underlying emotional triggers for smoking.

We hope that the presented findings can serve as a foundation for developing more effective clinical interventions, culturally sensitive public health strategies, and social policies aimed at curbing the global tobacco epidemic. By complementing physiological and epidemiological data with psychological variables, the framework for understanding and combating tobacco use becomes more robust. This integrative approach offers a more holistic understanding of the factors influencing tobacco use, paving the way for interventions that are both more effective and culturally sensitive.

## Limitations

While our study offers valuable insights, it is essential to consider these findings in the context of broader health behaviors and across different age groups. We acknowledge that the reported results are based on a sample of 50+ years of age adults. This limits the generalizability of our findings, as the personality profiles associated with smoking in older adults may differ from those in younger individuals, who are at the formative stages of establishing smoking habits. Future research should explore these relationships in younger cohorts, potentially informing early intervention strategies that preempt the onset of smoking based on personality predispositions.

Further studies could also expand the scope to include other forms of tobacco products such as chewing tobacco or more recent smoking trends such as e-cigarettes and vaping [37]. Broadening the scope of and differentiating between different forms of tobacco consumption would provide a more comprehensive view of modern smoking habits and a more targeted and hopefully more successful rate of smoking cessation programs. We acknowledge the value of examining this issue across age cohorts and inclusive of modern nicotine delivery preferences.

Finally, the reliance on self-reported data for personality traits and smoking behavior could also have introduced biases and inaccuracies. This highlights the need for longitudinal research to establish causality and explore the dynamic nature of smoking behavior over time.

## Conclusion

In conclusion, this study enriches our understanding of the psychological dimensions of tobacco use, providing a foundation for developing more precise and effective public health interventions. It is our hope that understanding the interplay between these factors can offer valuable insights for public health interventions aimed at reducing smoking rates and improving public health outcomes.
